# Human genetics and epigenetics of alcohol use disorder

**DOI:** 10.1172/JCI172885

**Published:** 2024-08-15

**Authors:** Hang Zhou, Joel Gelernter

**Affiliations:** 1 Department of Psychiatry, Yale School of Medicine, New Haven, Connecticut, USA.; 2 Veterans Affairs Connecticut Healthcare System, West Haven, Connecticut, USA.; 3 Department of Biomedical Informatics and Data Science,; 4 Center for Brain and Mind Health,; 5 Department of Genetics, and; 6 Department of Neuroscience, Yale School of Medicine, New Haven, Connecticut, USA.

## Abstract

Alcohol use disorder (AUD) is a prominent contributor to global morbidity and mortality. Its complex etiology involves genetics, epigenetics, and environmental factors. We review progress in understanding the genetics and epigenetics of AUD, summarizing the key findings. Advancements in technology over the decades have elevated research from early candidate gene studies to present-day genome-wide scans, unveiling numerous genetic and epigenetic risk factors for AUD. The latest GWAS on more than one million participants identified more than 100 genetic variants, and the largest epigenome-wide association studies (EWAS) in blood and brain samples have revealed tissue-specific epigenetic changes. Downstream analyses revealed enriched pathways, genetic correlations with other traits, transcriptome-wide association in brain tissues, and drug-gene interactions for AUD. We also discuss limitations and future directions, including increasing the power of GWAS and EWAS studies as well as expanding the diversity of populations included in these analyses. Larger samples, novel technologies, and analytic approaches are essential; these include whole-genome sequencing, multiomics, single-cell sequencing, spatial transcriptomics, deep-learning prediction of variant function, and integrated methods for disease risk prediction.

## Introduction

Alcohol use disorder (AUD) is a chronic relapsing disorder that progresses through a three-stage addiction cycle involving neurocircuitry in the basal ganglia, extended amygdala, and prefrontal cortex (1). Different terminologies and diagnostic approaches have been applied over the decades to AUD and related traits, including alcohol dependence and alcohol abuse based on DSM-IV (2) and prior editions and AUD based on DSM-5 (3). Diagnostic criteria include tolerance to the effects of alcohol; withdrawal in the absence of alcohol use; inability to control or reduce alcohol intake; preoccupation with alcohol to the detriment of work, family, and social priorities; and others (4). In addition, ICD diagnostic codes are widely used in clinical settings and reflected in electronic health records to diagnose AUD and related disorders. If not otherwise specified, henceforth, we use a broad definition of AUD to encompass both alcohol dependence and alcohol use disorder.

AUD and excessive alcohol use contribute greatly to the global disease burden and causing substantial adverse health effects (5). However, only three medications (disulfiram, naltrexone, and acamprosate) are approved by the US FDA for treating AUD (4). Thus, a lack of treatment options persists despite the clinical importance of the problem.

## Genetics of AUD: candidate genes and linkage studies

AUD is a complex disorder with significant environmental and genetic components. Genetic influences on AUD have long been established (6–8), and family and twin studies have reported about approximately 0.50 (95% CI, 0.43–0.53) genetic heritability (9–13). Three waves of genetic studies conducted in the past decades have identified susceptibility genes (14–18). The first wave involved candidate gene studies. In this Review, we omit from discussion underpowered studies. Candidate genes related to ethanol metabolism were intensively investigated for associations with AUD (19–25). Alcohol dehydrogenases (ADHs), such as *ADH1B* and *ALDH2,* are enzymes that oxidize ethanol into acetaldehyde, and aldehyde dehydrogenases are enzymes that catalyze aldehydes to their corresponding acids. The importance of functional variants such as rs1229984 (encoding His48Arg) in *ADH1B*, and rs671 (encoding Glu504Lys) in *ALDH2* is well-established. The increased catalytic efficiency of *ADH1B* (conferred by the His48 allele) or lower activity of *ALDH2* (by the Lys504 allele, which is common exclusively in East Asians) leads to accumulation of acetaldehyde and flushing (26, 27), which discourages further alcohol intake, thus protecting against AUD (28–31). Another coding variant, ADH1B*rs2066702 (Arg369Cys), has been associated with AUD but only in African populations (unless specified otherwise, the African ancestry samples in studies mentioned in this article were African-Americans) as it is nonpolymorphic in other populations (32, 33). Numerous additional candidate genes have failed to survive the GWAS era, and these will not be discussed here.

The second wave of genetic studies involved linkage studies, which utilize family data to identify genomic regions associated with AUD (34, 35). This wave was followed by positional candidate gene studies to target the relevant genes or variants. Several candidate genes have been identified using linkage analyses, including genes that encode GABA receptors (*GABRA1* and *GABRA2*) (36–39), *CHRM2* (cholinergic receptor muscarinic 2) (40, 41), and others. However, these genes were not identified in later GWAS.

## GWAS of AUD

**Figure 1:**
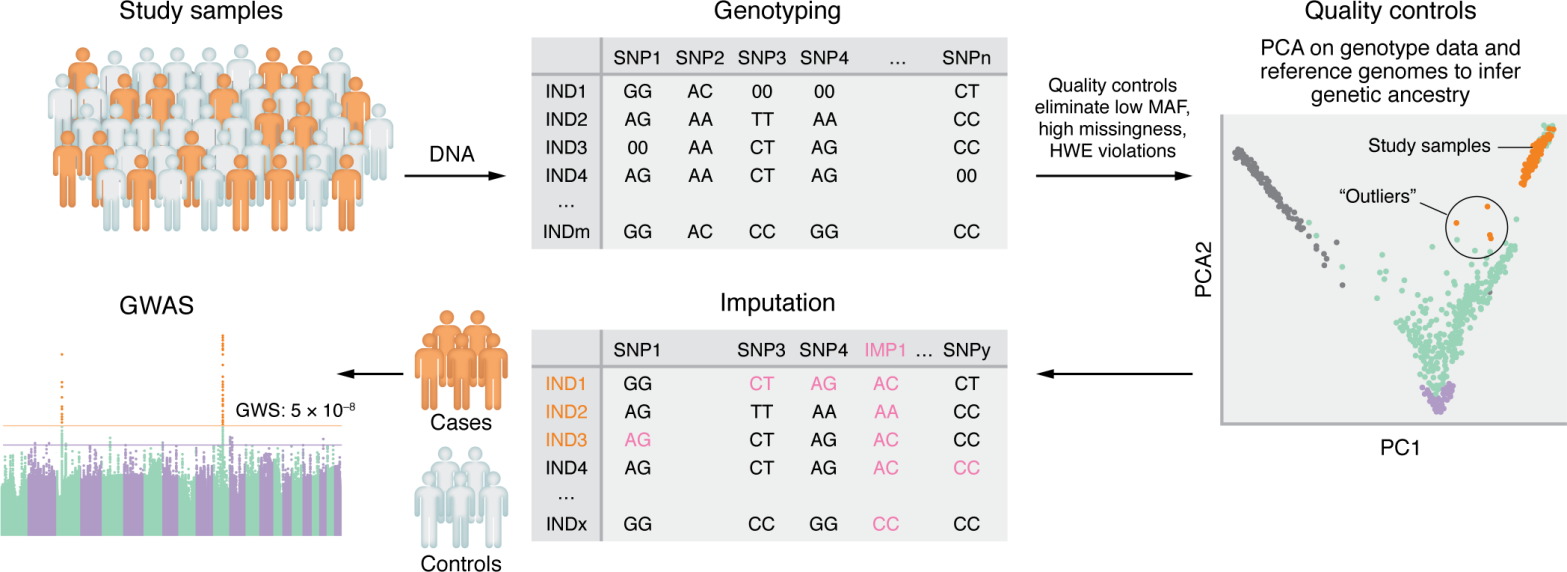
Workflow of GWAS. In a typical GWAS study, participants are recruited and provide written informed consent and blood or saliva samples for DNA extraction and genotyping using microarray (“00” indicates missing genotype call). Basic quality controls are performed to remove SNPs with low minor allele frequencies (MAF), high genotype missingness rate, or violation of Hardy Weinberg Equilibrium expectations (HWE) and remove samples with high genotype missingness. Since genetic factors often differ according to ancestry, principal component analysis (PCA) is performed on the data after quality controls with reference genomes — for example, the 1000 Genomes Project (165) — to infer the genetic ancestries of the study samples and remove genetic outliers (the results from different ancestry groups can then be combined by meta-analysis). Then, the remained samples and the data after quality control are imputed for millions more variants (imputed genotypes and SNPs [IMP], labeled in purple) using reference genomes (165–168). Imputation takes advantage of known patterns of linkage disequilibrium to provide useful data for many more variants than are genotyped directly. A study trait, in the context of either case-control status (for example, AUD) or continuous measurement (for example, AUD criterion counts), is assessed in the cohort. Regression models implemented in computational tools (169–175) are applied to test the association between each variant and the studied trait within the genetically inferred population group, adjusting for covariates including age, sex, and the top principal components of ancestry. Variants with *P* < 5 × 10^-8^ are considered genome-wide significant (GWS) after multiple testing corrections for the number of independent genomic regions evaluated (176).

**Figure 2:**
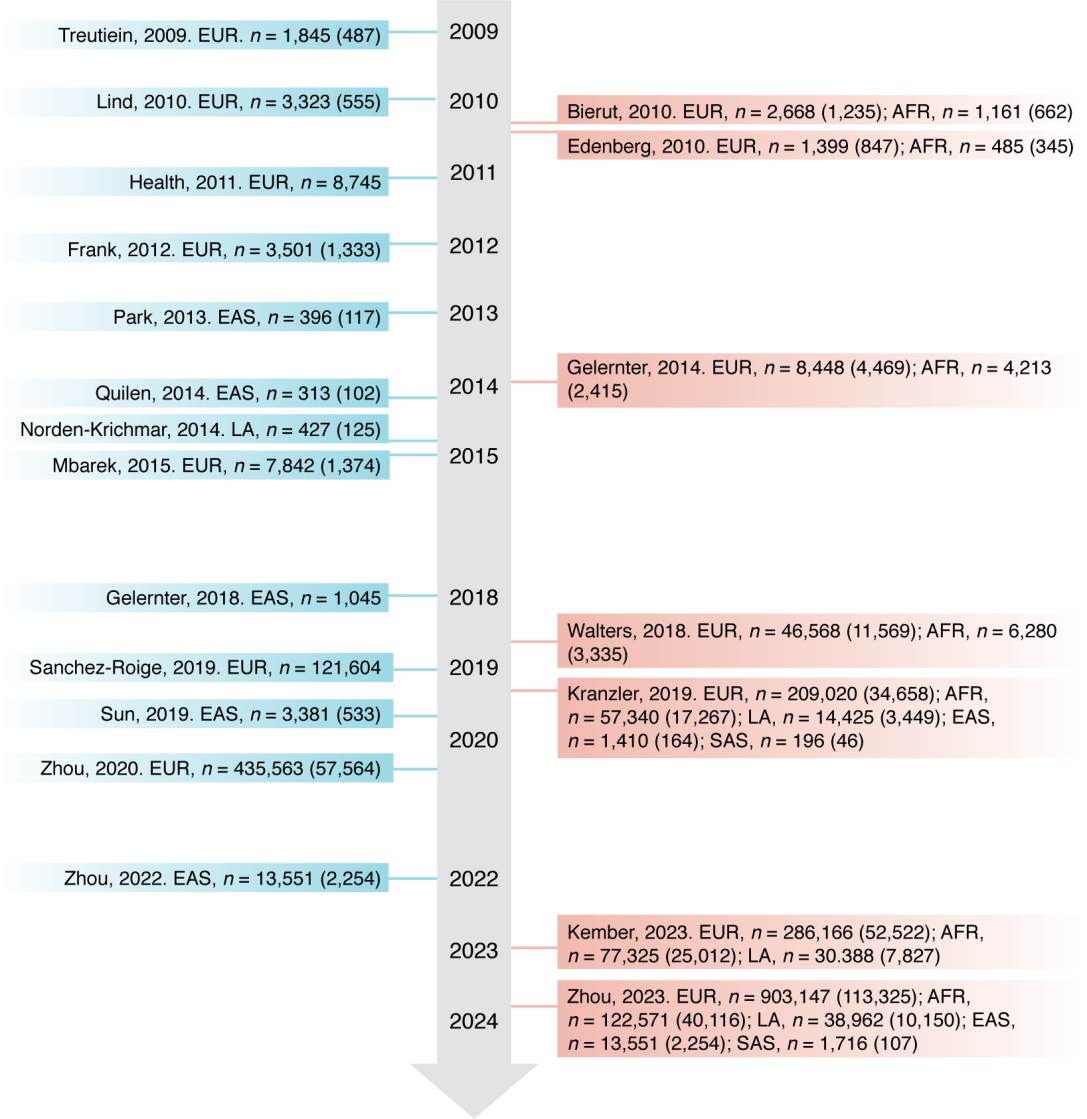
Timeline of the GWAS of AUD. Only studies with new samples are included here. Note that Sanchez-Rogie et al. (72) presented here is the GWAS of AUDIT-P in the UK Biobank. Studies with multiple ancestries are listed on the right. Numbers in the brackets are numbers of AUD cases included in the study. Table 1 highlights these studies in more detail. EUR, European; AFR, African; EAS, East Asian; LA, Latin American; SAS, South Asian.

Different from the previous candidate-gene studies, GWAS is a hypothesis-free method that scans genome-wide common variants using microarray genotyping or sequencing to identify associations with study traits (Figure 1) (42, 43). Substantial progress has been made in the wave of genetic studies of AUD using GWAS (Figure 2 and Table 1).

In 2009, the first GWAS of AUD was conducted in a German sample comprising 487 cases of AUD and 1,358 population-based controls; no variants reached the genome-wide significant (GWS) threshold (44). In 2011, the same team augmented the sample size by recruiting more participants and identified a variant located between *ADH1B* and *ADH1C*. In this study, the polygenic risk score (PRS, a method that quantifies an individual’s genetic predisposition to a particular trait or disease by summing the effects of multiple genetic variants across the genome) for AUD was investigated for the first time to test the association with AUD in independent samples, including splitting the study samples into two halves randomly, plus two samples from the National Library of Medicine’s Database of Genotypes and Phenotypes (45). In 2010, a study of Dutch and Australian samples was the first AUD GWAS to apply imputation for missing SNPs using the HapMap reference panel (46). The study reported no GWS results for AUD, but three SNPs were identified for comorbid AUD and nicotine dependence (47). No association was identified in a general community sample in Australia, but this study discussed the polygenic nature of AUD and projected the need for larger sample size (48). Reanalyses of these cohorts were undertaken to enhance statistical power (49–52).

Insufficient degree of genetic diversity in study populations has been a persistent challenge in human genetic studies, with the majority of study participants being of European ancestry (53, 54). Including non-European populations in AUD GWAS could help illuminate the shared and specific genetic architectures across populations. Three GWAS of AUD extended the gene discovery effort to more populations (55–57). However, no GWS signals were identified in these studies. Subsequently, several GWAS of AUD were performed of East Asian samples. The first was a study of a Korean sample with 396 unrelated individuals, which identified both the ADH1B*rs1229984 and ALDH2*rs671 (58). Thus far, the well-known functional coding variants rs1229984 and East Asian–specific rs671 have been confirmed by the GWAS approach. Other studies also identified the *ALDH2* region to be associated with AUD in East Asian samples (59–62), with no additional risk variants identified beyond these two regions. Meta-analyzing (a method that combine GWAS results from two or more separate cohorts) newly recruited samples with previously published summary data provides an opportunity to uncover additional risk variants. In 2014, a study involving more than 10,000 individuals of African and European ancestries was conducted, combining several cohorts. In both case-control analysis and criterion-count analysis, the ADH gene region was confirmed, and a strong association with the coding variant rs2066702 (Arg369Cys) in *ADH1B* was identified in African samples. Four other loci were associated with AUD in the criterion-count analysis: two in European ancestry samples and two in African ancestry samples (63). A large meta-analysis of AUD from the Psychiatric Genomics Consortium combined 28 studies of individuals of both European (*n* = 46,568) and African (*n* = 6,280) ancestries, confirming associations with the ADH gene cluster; however, no additional risk variants were discovered (64). This study also investigated the genetic correlations between AUD and many other traits, observing significant correlations with psychiatric disorders, substance use traits, and socioeconomic status (educational attainment and Townsend deprivation score). PRS derived from the European GWAS showed weaker predictions in independent African sample than the PRS derived from African GWAS, indicating limited portability of PRS across ancestries (65).

Besides DSM or ICD diagnosis, AUD can be assessed using the Alcohol Use Disorders Identification Test (AUDIT), a 10-item questionnaire developed by the WHO to measure hazardous or harmful drinking in the past year (66). Questions 1–3 are aimed at assessing alcohol consumption levels (AUDIT-C), and questions 4–10 are focused on evaluating problematic alcohol drinking (AUDIT-P). The AUDIT is useful to screen for AUDs (67, 68). Thus, such a questionnaire could be implemented as a cost-effective strategy for phenotyping samples in large-scale cohorts or biobanks.

The first two GWAS of AUDIT scores identified no association (69, 70). A later study of AUDIT in two population-based cohorts, the UK Biobank (71) and 23andMe (69), totaling 141,932 participants, identified 15 independent signals in 11 genomic loci for AUDIT total score, many of them novel (72). Four loci were associated with AUDIT-P subscore, including the ADH region, *KLB* (encoding β-klotho), and *SLC39A8* (solute carrier family 39 [zinc transporter], member 8). Another key finding from this study is that the genetic architecture of AUDIT-P differed from AUDIT-C, and AUDIT-P is genetically correlated with AUD more strongly than AUDIT-C. Transcriptome-wide association study (TWAS) (73) identified 26 genes whose predicted gene expression in brain tissues were associated with AUDIT.

A study in the Million Veteran Program (MVP) (74) investigated both AUD and alcohol consumption (measured by AUDIT-C) in five population groups, including European, African, East Asian, Latin American, and South Asian populations (75). This study included 274,391 participants, with 55,584 diagnosed with AUD based on the ICD codes. Fifteen independent variants (after conditional analyses) in 10 loci were identified in multiple ancestries, including 10 in European, 2 in African, and 2 in Latin American ancestries. Partitioning heritability analysis to investigate how the cell type–specific functional categories of the genome contribute to the heritability of a complex disease (76) indicated that the CNS was the most significantly enriched cell type group for AUD, confirming with genetic evidence that AUD is a brain-related disorder. This study also delivered a key finding that the genetic architecture of alcohol consumption (measured by AUDIT-C) differs from that of AUD (a similar pattern was observed between AUDIT-C and AUDIT-P, ref. 72), stressing that analyzing AUD or AUDIT-P separately from alcohol consumption traits would reduce heterogeneity. Prior to these two key papers, it was not recognized that quantity/frequency versus dependence measures differed genetically and therefore biologically. Another cross-ancestry study used longitudinal data from MVP and confirmed this difference between AUD and AUDIT-C and identified novel loci with both traits. Specifically, this study identified a set of variants with effects on AUD that are not mediated through alcohol consumption (i.e., AUDIT-C) (77).

A subsequent study of problematic alcohol use (PAU), a proxy phenotype of AUD, combined AUD from the MVP and Psychiatric Genomics Consortium and AUDIT-P from UK Biobank and identified 29 independent risk variants in 435,563 EUR participants (78). In this study, the genetic correlation between AUDIT-P and AUD was estimated to be 0.71 (standard error = 0.05), justifying the proxy-phenotype meta-analysis of PAU across these data sets. This study noted the heterogeneity among these phenotypes and discussed that associations specific to each definition could have been attenuated. A total of 327 known drug-gene interactions were found for 16 associated genes, with *DRD2* having the most drug interactions (*n* = 177) followed by *BDNF* (*n* = 68) and *PDE4B* (*n* = 36). Phenome-wide PRS analysis in the independent biobank BioVU confirmed the genetic correlations between PAU and substance use and psychiatric disorders. Pathways including reactome ethanol oxidation and ethanol and alcohol metabolism were the most significantly enriched for AUD. TWAS showed significant enrichments in several brain tissues, including the cerebellum and cortex, further illustrating the tissue-specific mechanisms of this brain-related disease. Mendelian randomization analysis (79), a set of methods that uses genetic variants as instrumental variables to estimate the causal relationship between exposure and outcome, suggested liability to substance use, psychiatric status, risk-taking behavior, and cognitive performance having causal effects on the liability to PAU.

## Findings of the 2023 multiancestry GWAS of PAU

Thus far, studies have identified risk genes associated with AUD in multiple ancestries and have repeatedly confirmed the associations of several genes, mostly in populations with European ancestry. In 2023, a multiancestry study of PAU with more than 1 million participants revealed numerous novel findings (80). 85 independent risk variants were identified in participants with European ancestry, and 110 risk variants in total were identified in either within-ancestry or cross-ancestry meta-analysis. Cross-ancestry fine-mapping identified credible sets in 13 loci (a set of plausible causal variants within each locus — these sets of putative causal variants are called “credible sets”) containing a single variant. There were 34 additional credible sets containing 2–5 variants. Taken together, these results provided a list of target variants for future experimental functional studies. Leveraging information from multiple ancestries, the cross-ancestry PRS association findings were greater than those using single-ancestry PRS (81).

This study examined overlapped genes by both gene-based association analysis and TWAS (82) in brain tissues and/or chromatin interaction analysis (83) using Hi-C brain annotations. Many genes showed convergent evidence linking association to PAU with brain biology through gene expression (TWAS) and chromatin interaction (Hi-C) analyses. Translating genetic results into clinical applications is an important goal of human genetic studies, and previous studies have demonstrated the possibilities (84–86). Through two types of drug-repurposing analyses, this study identified existing medications as potential treatments for AUD. The first analysis searched the independent genetic signals in Open Targets (87) for druggability and medication target status. Many genes were druggable, including *DRD2*, *CACNA1C*, *DPYD*, *PDE4B*, *KLB*, *BRD3*, *NCAM1*, *FTO*, *MAPT*, *OPRM1*, and *GABRA4*. The second drug repurposing analysis, using TWAS results, found that 287 compounds were significantly correlated with the transcriptional pattern associated with risk for AUD. These compounds include trichostatin-a, melperone, triflupromazine, spironolactone, amlodipine, and clomethiazole. Trichostatin-a has effects on preventing the development of alcohol withdrawal-related anxiety in rats (88), clomethiazole is used to treat alcohol withdrawal syndrome (89), and spironolactone reduces alcohol use in both rats and humans with convergent evidence (90). This study provided a list of potential medications and targets for future pharmacological studies for AUD.

## Limitations of AUD GWAS and future directions

While the field of AUD genetics has made considerable progress, substantial gaps persist (similar to other psychiatric disorders, ref. 91). Here, we highlight some limitations of the current AUD studies (Table 2) with the hope that gaps may be filled with new data sets, technologies, analytic methods, and research directions in the future.

(a) Different definitions of AUD and proxy phenotypes (e.g., AUDIT-P) have shared genetic architecture, resulting in improved power in gene discovery when they are combined from different cohorts (78, 80). However, they are not identical traits. Deep phenotyping (either using same definition or focusing on subphenotypes) in larger cohorts could reduce the phenotypic heterogeneity and increase the possibility of identifying trait-specific associations and pathways (92).

(b) Some studies have endeavored to include samples in multiple ancestries (55, 56, 63, 64, 75, 80), but the sample sizes in the non-European ancestries are smaller than sample sizes in the European ancestries — a common issue in human genetic studies (53, 54). Recruitment of individuals of diverse genetic ancestries is a critical next step in this field. With more multiancestral biobanks becoming available, including MVP, the Global Biobank Meta-analysis Initiative (93), and the All of Us Research Program (94), we anticipate that the gap in diversity will diminish. Funding agencies should also direct attention to studies that propose recruitment focused on non-European ancestry participants.

(c) AUD is a highly polygenic disorder, with hundreds of variants at least contributing to the risk (80, 95). The “brute force” GWAS approach requires a larger sample size to identify more risk variants. Unlike other traits or behaviors that can be measured directly and assessed in large populations or biobanks — for example, GWAS of height (96), educational attainment (97), and alcohol consumption (98) have been conducted in 3~5 million participants — clinical diagnosis of AUD in large cohorts is still lagging. Similar to point (a), increasing sample size and incorporating multiple ancestries could improve the power and resolution of causal variant fine-mapping (80). Besides the well-known functional coding variants in the alcohol metabolic genes, most variants identified through large GWAS have small to very small effects on the risk of AUD, reducing the yield of the extensive effort of following functional studies on individual variants. This is a common issue in the genetic study of complex traits.

(d) Current GWAS studies have mostly used SNP arrays and post hoc imputation to fill in common variants, which does not allow analysis of the full genome because some parts of the genome are not fully “covered” — i.e., there are unassessed variants in some genomic regions that cannot be tested for association, for technical reasons. A typical SNP array can capture from 600,000 (for example, Illumina PsychArray) to 1.8 million (for example, Illumina Multi-Ethnic Genotyping Array) variants. After imputation and application of standard quality controls for the variants, typical analyzable numbers of high-quality variants vary from 5 to 15 million, depending on the original array SNP density, sample size, and genetic ancestry (from a population genetics point of view, African populations have more common variants than other populations due to their evolutionary history). Given the inherent missing information from different steps, GWAS meta-analyses can only cover a subset of variants of the whole genome, indicating that much of the genome is missing in the current genetic studies of AUD. Whole-genome sequencing (WGS), which can detect essentially all variants (including rare variants and structural variants) without ascertainment bias, could provide better opportunities to investigate the full genetic architecture of the trait.

Several whole-exome sequencing (WES) studies and one WGS study of AUD have been conducted recently (99–102). A phenome-wide WES study of 170,979 individuals (6,320 cases) from the UK Biobank identified two common variants in the *ADH1C* gene associated with AUD (*P* < 2 × 10^–9^), using either an additive or a dominant model (102). A WES study combining 469,835 individuals from the UK Biobank data (13,121 cases) and 3,789 individuals from the Yale-Penn cohort (2,562 cases) with multiple ancestries identified the well-known functional variant ADH1B*rs1229984 and several common variants in *ADH1C*. Gene-based tests accounting for the burden from loss-of-function, missense, and synonymous variants identified novel genes *CNST* and *IFIT5* (101). A low-coverage WGS study of AUD-related life events and two affective symptoms in 742 American Indians and 1,711 European Americans identified both common and rare novel variants (103).

(e) Most variants identified by GWAS are in noncoding regions with unknown functions (104). The top associated variants in each risk locus are not necessarily the causal variants for AUD. Although post-GWAS fine-mapping analysis could identify a credible set of potential causal variants (105–107), further efforts are needed to interpret and validate the variants’ functions. In recent years, novel analytic approaches like deep learning (a subset of machine learning) have been successfully implemented in biomedical research. For example, deep-learning methods contribute to prediction of protein structure (108, 109), pathogenic missense variants (110, 111), and regulatory functions of genome variations (112–115). Combining novel computational tools and cutting-edge functional essays like genome editing (116–118) could help assess the variants’ effects at scale.

(f) Although hundreds of risk variants have been identified and many have been repeatedly replicated in GWAS, indirect genetic effects (also called “genetic nurture”), which are effects of alleles in parents on offspring through the environment (119), have not been distinguished from direct genetic effects on AUD. Methods have been developed to impute parental genotypes using family data (120), which could be used to improve estimates of direct genetic effects for AUD. Confounding effects, including socioeconomic status, may also bias the results. For example, educational attainment influences many psychiatric and nonpsychiatric traits (97) and has a genetic correlation *r*_g_ = –0.21 with AUD, which needs to be considered in future studies.

(g) Another profound gap is that the current predictive performance of PRS for AUD based on GWAS common variants — i.e., using genetic variation to predict risk in genotyped individuals — is strongly statistically significant but numerically still weak and has not yet entered the range of clinical utility. Despite the increase in sample size, the SNP-based heritability (*h*^2^) by GWAS is low (*h*^2^ ranges from 5.6% to 12.7% with liability-scale *h*^2^ ranging from 8.9% to 16.2%, refs. 64, 72, 75, 78, 80) compared with the total heritability but comparable to what is observed for many other genetically complex traits. PRS presently has limited power for AUD prediction (explained variance measured by pseudo *R*^2^) in independent cohorts; thus, the clinical use of the current PRS of AUD is not imminent. Possibly, the success of artificial intelligence in other areas could extend into predicting AUD risk, with more genomic and large-scale electronic health records data available by integrating improving genomic data with other trait predictors.

(h) Finally, genetic studies have confirmed that AUD is partly a brain-related disorder (75). Genes with expression perturbation in specific brain tissues have been prioritized (72, 80), but the biological pathways from genetics to the etiology of AUD are largely unclear. There are major exceptions though: the mechanism of the effect on risk of alcohol-metabolizing enzyme variation is well understood. Many biological processes play roles in the pathways, such as gene expression, functional regulation, protein perturbation, metabolites, and other mediating traits. To understand the pathway mechanisms, studies beyond genetics are warranted, including, but not limited to epigenetics (discussed in *Epigenetics of AUD*), multiomics, single-cell sequencing, and the latest spatial transcriptomics.

## Epigenetics of AUD

Epigenetic studies of AUD have emerged as an important avenue for understanding the complex interplay among genetics, environment, and gene regulation in the development and progression of AUD. Epigenetic factors include transcription factors, noncoding RNAs, DNA modifications, or histone modifications that alter the gene expression and consequently affect phenotypes, without changing the DNA sequence (121, 122). While epigenetic status is highly heritable and affected by environmental factors, including alcohol exposures, certain epigenetic changes in specific brain regions have been implicated in the etiology of AUD (123).

Although most epigenetic studies in humans have focused on alcohol consumption (which is not the main focus of this Review; we focus here on use *disorder* rather than use), some studies have explored DNA methylation patterns in individuals with AUD and identified differential methylation in specific genomic regions (reviewed in refs. 124–128). These changes are often observed in genes related to neurobiological processes, neurotransmitter systems, and immune responses. For example, significantly greater DNA methylation in the *HERP* promoter was reported in patients with AUD than controls (129), while a higher level of DNA methylation in the promoter region of the *OPRM1* gene was observed in AUD (130). A study of postmortem human brains found an overall decrease in methylation in the long-terminal repeat retrotransposons in the frontal cortex (131). Notably, these brain samples, along with those used in several follow-up epigenetic studies of AUD, were mostly from the New South Wales Tissue Resource Centre (NSW TRC) at the University of Sydney (132). However, no global methylation differences were observed between AUD cases and controls in the frontal cortex (133). DNA hypermethylation was also reported in other genes, including *SNCA* (134), *MAOA* (135), *DAT* (136), *NGF* (137), *AVP* (138), *PDNY* (139), and *GABRD* (140). In a study of 285 African Americans and 249 European Americans using a custom-designed methylation array of 384 CpGs in 82 candidate genes, a significant CpG site was identified in the *HTR3A* (5-hydroxytryptamine receptor 3A) promoter region in European Americans. Several other suggestive CpGs were also reported in either African Americans (in genes *GABRB3* and *POMC*) or European Americans (in genes *NCAM1*, *DRD4*, *MBD3*, *HTR2B*, and *GRIN1*) (141). As for studies of genetic variation, it is unclear whether “candidate gene” results will be proven stable over time in epigenetic studies.

## Epigenome-wide association studies of AUD

Most DNA methylation studies in AUD have focused on individual gene regions and did not produce replicable results; epigenome-wide scans are needed to identify AUD-related epigenetic changes at scale. Similar to the waves of technologies in genomic studies, microarrays and next-generation sequencing techniques have been applied to epigenetic studies of AUD (Table 3).

The first epigenome-wide association study (EWAS) on AUD involved peripheral blood samples from 10 AUD cases and 10 controls of East Asian ancestry. In total, 865 hypomethylated and 716 hypermethylated CpG sites were identified (defined as expression difference score ≥20) (142). The second study involved a cohort of 128 East Asian males, with 63 individuals diagnosed with AUD. In this study, significantly lower levels of methylation were observed in cases compared with controls, with 1,702 hypomethylated and 8 hypermethylated sites reaching FDR *P* < 0.005 (143). A study of 33 patients from alcohol treatment centers and 33 individuals acting as healthy controls (abstinent from alcohol for six months) — mostly European males — identified 56 differentially methylated CpG sites. None of these sites remained significant after the 30-day inpatient treatment program (144). A longitudinal study of East Asian samples identified 149 hypermethylated and 51 hypomethylated genes (*P* < 0.01) between healthy (1990–1992) and dependent phases (2003–2009) in 10 individuals (145). Given the changes in methylation status with the change of alcohol use status in the cases, further studies of AUD with a more robust experiment design that eliminates the ongoing effects of alcohol use are warranted. Similar patterns were observed in a larger longitudinal study of 99 in-patient AUD cases and 95 matched individuals acting as controls. Blood samples were collected in two phases from the affected participants, one during acute alcohol withdrawal and the other after two weeks of recovery in the treatment centers. Compared with the controls, 9,845 CpGs were identified during alcohol withdrawal and 6,094 after two weeks. Comparing the two phases within cases revealed 2,876 differentially methylated CpG sites, suggesting reversibility of alcohol- and withdrawal-related methylation (146). EWAS in 18 discordant monozygotic twin pairs, i.e., one affected and one not in each pair, identified 77 differentially methylated regions at FDR < 0.05 (147). A larger EWAS of AUD in 539 blood samples reported 5,101 significant differentially methylated CpG sites after FDR correction. Of these, 96 CpG sites were replicated in a second cohort of 43 AUD cases and 43 controls (148).

The studies mentioned above were conducted primarily on blood samples, with later investigations focusing on postmortem brain samples from NSW TRC to identify DNA methylation changes associated with AUD. A study specifically analyzed prefrontal cortex samples from 23 AUD cases and 23 controls, with a particular emphasis on sex-stratified analyses due to a previous observation of a sex-biased methylome (149). Among 32 males (16 AUD cases), 1,201 hypermethylated and 611 hypomethylated CpG sites were discovered at an FDR level of 0.05; however, no significant results were observed in females (150). Reanalysis of the same data, combining both sexes, identified three CpG sites after FDR-based correction (151). EWAS of 49 AUD cases and 47 controls using the 450,000 methylation array identified 561 hypomethylated CpGs and 485 hypermethylated CpGs were reported with *P* < 1 × 10^–7^ (152). A small EWAS on brain samples from 23 AUD cases and 23 controls, using a higher density methylation array (850,000 CpG sites) found no GWS loci (see Figure 1C in ref. 153), although there were 1,218 CpGs with *P*_nominal_ ≤ 0.001 (153).

Several studies investigated multiple brain regions simultaneously. For instance, a study of two brain regions, including the prefrontal cortex and nucleus accumbens in 86 individuals, did not identify single CpG sites but identified two differentially methylated regions (permutation *P* < 0.05) mapping to the upstream regions of *ZFP57* and *DLGAP2* genes (154). An EWAS on five brain regions from 111 individuals identified two differentially methylated CpG sites in the caudate nucleus region and 18 in the ventral striatum, with no significant findings in the other three regions (155). Despite utilizing brain samples from the same repository (NSW TRC), the reported findings across these studies were largely inconsistent.

In an additional study on AUD, brain samples from 119 individuals from the Lieber Institute for Brain Development Human Brain Repository were analyzed. This investigation identified 53 CpGs associated with AUD in the nucleus accumbens and 31 in the dorsolateral prefrontal cortex (BA9) at FDR *P* < 0.05, with no overlap across the two regions. The investigators conducted a meta-analysis across the two regions, revealing an additional 21 CpGs, bringing the total to 105 unique AUD-associated CpGs in 120 genes (156). Comparing the results of this study with previous research on brain regions (152, 155, 157), only one intergenic CpG, cg00402668 in the BA9, reached a “look-up” level of significance (FDR *P* < 0.05) in BA10 from Clark et al. (157), but not in other brain regions. On the gene level, three annotated genes in this study overlapped with genes from Hagerty et al. (152). When testing for overlap of the top 1% of CpG sites across studies, a significant enrichment for the nucleus accumbens results in this study was observed in the putamen and ventral striatum results from Zillich et al. (155).

Cross-tissue studies have advanced the understanding of overlapping epigenetic mechanisms. In a study of 1,132 blood samples consisting of four cell types and 50 brain samples, over 21 million CpG sites were assessed using methyl-CG binding domain sequencing. No significant associations were observed for the whole blood or brain. However, for T cells and monocytes, 3 CpGs and 1,397 CpGs were identified at FDR *P* < 0.1, respectively. One CpG site in the *DLGAP1* gene was significantly replicated, and an additional 34 sites were nominally replicated in an independent sample of 73 AUD cases and 339 controls. Beyond DNA methylation, this study also assessed hydroxymethylation for over 26 million CpG sites in the brain samples. While no individual site reached methylome-wide significance, the study observed significant overlap between the top sites in blood cell type–specific EWAS and both methylation and hydroxymethylation EWAS in the brain. This represents the first exploration of cell type–specific methylation for AUD in blood and considers the role of brain hydroxymethylation in AUD (157).

## Other epigenetic studies of AUD

Besides studies of DNA methylation for AUD, limited studies of noncoding RNAs and histone modifications in the human brain have been conducted. microRNA (miRNAs) are small noncoding RNAs that regulate target mRNA expression and/or translation, with important roles in a variety of biological processes (158). A study of miRNAs in the prefrontal cortex of 27 individuals identified 12 upregulated miRNAs (FDR *P* < 0.05) in AUD cases (*n* = 14) compared with controls (*n* = 13), suggesting a regulatory role of miRNAs in AUD gene expression (159). A candidate gene study of long noncoding RNA BDNF-AS in the human amygdala implicated a regulatory effect on *BDNF* expression in early-onset (before age 21) AUD cases (*n* = 11) compared with controls (*n* = 22) but not late-onset AUD (160).

A study of the transcriptome using RNA-Seq and histone H3 lysine 4 trimethylation (H3K4me3) using ChIP-Seq in postmortem brain hippocampus samples from the University of Miami Brain Bank identified 11 differentially expressed genes with FDR *P* < 0.05 in AUD cases (*n* = 8) compared with controls (*n* = 8). However, no H3K4me3 changes reached FDR *P* < 0.05 or overlapped with expression changes (161). Reanalyzing the data using network approaches identified 7 coexpression modules enriched for H3K4me3-associated changes in AUD cases compared with controls, suggesting relationships between this epigenetic mark and gene expression (162). The small sample size and low power indicate that these results should be taken with caution.

Another study investigated gene coexpression and its relationship with multiple epigenetic modifications for AUD in brain tissues from the NSW TRC. Central and basolateral nucleus of amygdala and superior frontal cortex from 17 AUD cases and 15 controls were assessed for gene expression, cortex from 6 AUD cases and 6 controls were assessed for global H3K4 methylation and DNA methylation of long-terminal repeat retrotransposons, and cortex from 5 AUD cases and 5 controls were assessed for histone H3K4me3 (ChIP-Seq). This study identified critical cellular components and previously unrecognized epigenetic determinants of gene coexpression relationships and discovered novel markers of chromatin modifications in the human brain (131).

In conclusion, epigenetic studies have provided limited insights into the molecular mechanisms underlying AUD. Considering the known genetic and etiologic complexity of AUD risk, and the contributions of both genes and environment, larger samples will be required to draw durable conclusions about AUD epigenetics. The integration of DNA methylation, histone modifications, and noncoding RNAs into our understanding of AUD pathogenesis holds promise for identifying novel therapeutic targets and developing personalized interventions. As technology advances and research methodologies are refined, the field of epigenetics is expected to profoundly contribute to unraveling the complexities of AUD. However, the field is presently limited greatly by a lack of well-powered investigations.

## Limitations of epigenetic studies in AUD

To date, there has been very limited progress in unraveling the epigenetic landscape of AUD. The heterogeneity of AUD, coupled with varying degrees of alcohol exposure in different groups and stages of the disorder, introduce complexities in interpreting findings, especially in small samples. Additionally, the dynamic nature of epigenetic modifications requires sophisticated study designs to capture temporal changes throughout AUD development. Comparatively, genetic studies of AUD, mainly through GWAS, have had much larger sample sizes (many orders of magnitude), while current epigenetic studies, particularly in the human brain, are small, with the largest study to date involving 119 individuals (156). Finally, the overlapping findings across tissues and studies are minimal (126, 156). While this could be attributed to tissue/context-specific epigenetic changes, it also raises the possibility of false-positive results.

As for GWAS of AUD, larger-scale epigenetic studies will be required to generate replicable findings and prioritize robust genomic variations for future pharmacological studies. Other future directions that have the potential to improve our understanding of the epigenetic mechanisms of AUD include the following. First, longitudinal studies tracking the epigenetic changes over time (163) could help us understand the temporal relationship between alcohol consumption and epigenetic modifications and work out the cause and effect on AUD. Second, integrating epigenetic data with other omics data (e.g., transcriptomics) should help us gain a better understanding of relevant regulatory mechanisms. Third, it is important to investigate how environmental factors (e.g., stress and diet) interact with epigenetic factors to influence the risk of AUD. Fourth, the current EWASs are focused on tissue-level changes; performing cell type–specific epigenetic analyses (e.g., single-cell epigenome data, ref. 164) can provide deeper insight into the molecular mechanisms.

## Conclusions

Genetic studies of AUD have greatly advanced our understanding of its complex etiology, while epigenetic studies have made limited progress. Although these studies have provided valuable insights, challenges and gaps in our comprehension persist, emphasizing the need for continued research and exploration incorporating larger samples with deeper phenotyping in more diverse populations. Integration of comprehensive insights from both genetic and epigenetic studies holds promise for the development of targeted and personalized therapeutic strategies, representing a crucial step forward in addressing the multifaceted nature of AUD. Future research should aim to increase statistical power, expand the study populations to encompass diverse groups, and thus refine our understanding of the mechanisms involved. Overcoming limitations and translating research findings into effective clinical interventions for AUD should be at the forefront of ongoing efforts.

## Figures and Tables

**Table 3:**
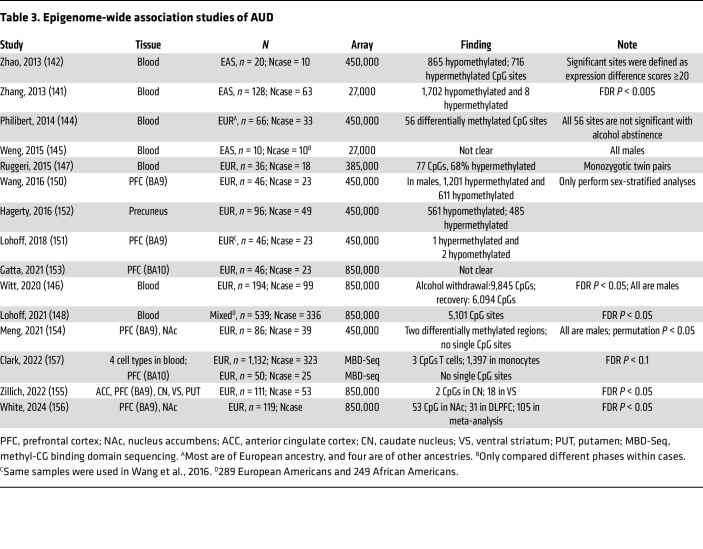
Epigenome-wide association studies of AUD

**Table 2:**
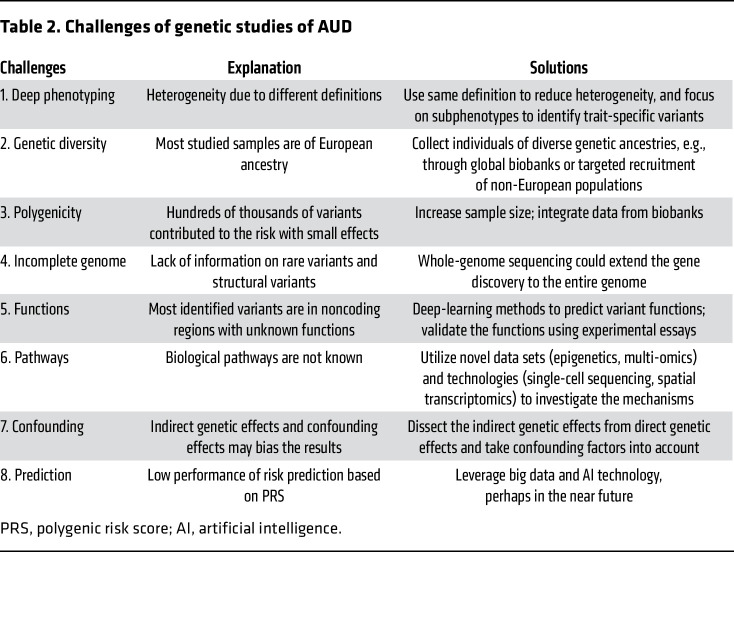
Challenges of genetic studies of AUD

**Table 1:**
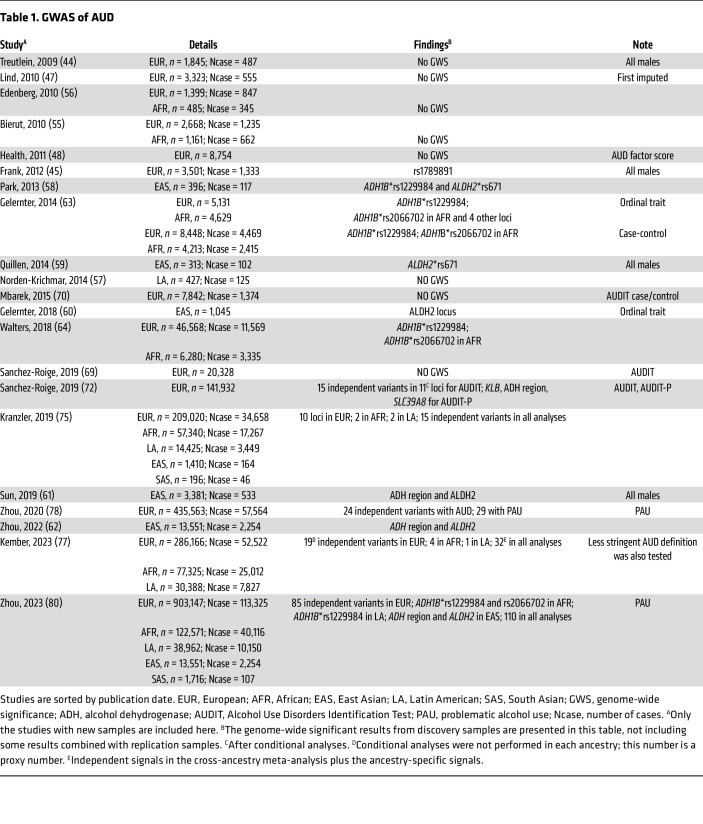
GWAS of AUD

## Version 1 08/15/2024

Electronic publication
